# Experience of the first adult-focussed undiagnosed disease program in Australia (AHA-UDP): solving rare and puzzling genetic disorders is ageless

**DOI:** 10.1186/s13023-024-03297-5

**Published:** 2024-08-02

**Authors:** Mathew Wallis, Simon D. Bodek, Jacob Munro, Haloom Rafehi, Mark F. Bennett, Zimeng Ye, Amy Schneider, Fiona Gardiner, Giulia Valente, Emma Murdoch, Eloise Uebergang, Jacquie Hunter, Chloe Stutterd, Aamira Huq, Lucinda Salmon, Ingrid Scheffer, Dhamidhu Eratne, Stephen Meyn, Chun Y. Fong, Tom John, Saul Mullen, Susan M. White, Natasha J. Brown, George McGillivray, Jesse Chen, Chris Richmond, Andrew Hughes, Emma Krzesinski, Andrew Fennell, Brian Chambers, Renee Santoreneos, Anna Le Fevre, Michael S. Hildebrand, Melanie Bahlo, John Christodoulou, Martin Delatycki, Samuel F. Berkovic

**Affiliations:** 1 Austin Health Clinical Genetics Service, Austin Health, Melbourne, Australia; 2 The Walter and Eliza Hall Institute of Medical Research, Parkville, Australia; 3 Department of Medical Biology, University of Melbourne, Parkville, Australia; 4 Epilepsy Research Centre, University of Melbourne, Austin Health, Melbourne, Australia; 5 Department of Paediatrics, Austin Health, Melbourne, Australia; 6 Victorian Clinical Genetics Service, Melbourne, Australia; 7 Murdoch Children’s Research Institute, Melbourne, Parkville, Australia; 8 Department of Paediatrics, The University of Melbourne, Parkville, Australia; 9 Tasmanian Clinical Genetics Service, Tasmanian Health Service, Hobart, TAS Australia; 10 School of Medicine and Menzies Institute for Medical Research, University of Tasmania, Hobart, Australia; 11 Genetics Service, Royal Prince Alfred Hospital, Melbourne, Australia; 12 Neuropsychiatry, The Royal Melbourne Hospital, Melbourne, Australia; 13 Genetics Service, Mercy Hospital for Women, Melbourne, Australia; 14 Neurology Service, Austin Health, Melbourne, Australia; 15 Royal Brisbane and Women’s Hospital, Brisbane, QLD Australia; 16 Medicine, Dentistry and Health Science, The University of Melbourne, Parkville, Australia; 17 Monash Health Genetics Clinic, Melbourne, Australia; 18 Genetic Medicine Service, The Royal Melbourne Hospital, Melbourne, Australia; 19 Peter MacCallum Cancer Centre, Melbourne, Australia; 20 Centre for Human Genomics and Precision Medicine, University of Wisconsin-Madison, Madison, WI USA

**Keywords:** Rare disease, Genome sequencing, Mosaicism, Genotype, Phenotype, Undiagnosed disease, Tuberous sclerosis, *TOP3B*, *PRKACB*, *NARS*

## Abstract

**Background:**

Significant recent efforts have facilitated increased access to clinical genetics assessment and genomic sequencing for children with rare diseases in many centres, but there remains a service gap for adults. The Austin Health Adult Undiagnosed Disease Program (AHA-UDP) was designed to complement existing UDP programs that focus on paediatric rare diseases and address an area of unmet diagnostic need for adults with undiagnosed rare conditions in Victoria, Australia. It was conducted at a large Victorian hospital to demonstrate the benefits of bringing genomic techniques currently used predominantly in a research setting into hospital clinical practice, and identify the benefits of enrolling adults with undiagnosed rare diseases into a UDP program. The main objectives were to identify the causal mutation for a variety of diseases of individuals and families enrolled, and to discover novel disease genes.

**Methods:**

Unsolved patients in whom standard genomic diagnostic techniques such as targeted gene panel, exome-wide next generation sequencing, and/or chromosomal microarray, had already been performed were recruited. Genome sequencing and enhanced genomic analysis from the research setting were applied to aid novel gene discovery.

**Results:**

In total, 16/50 (32%) families/cases were solved. One or more candidate variants of uncertain significance were detected in 18/50 (36%) families. No candidate variants were identified in 16/50 (32%) families. Two novel disease genes (*TOP3B*, *PRKACB*) and two novel genotype–phenotype correlations (*NARS,* and *KMT2C* genes) were identified. Three out of eight patients with suspected mosaic tuberous sclerosis complex had their diagnosis confirmed which provided reproductive options for two patients. The utility of confirming diagnoses for patients with mosaic conditions (using high read depth sequencing and ddPCR) was not specifically envisaged at the onset of the project, but the flexibility to offer recruitment and analyses on an as-needed basis proved to be a strength of the AHA-UDP.

**Conclusion:**

AHA-UDP demonstrates the utility of a UDP approach applying genome sequencing approaches in diagnosing adults with rare diseases who have had uninformative conventional genetic analysis, informing clinical management, recurrence risk, and recommendations for relatives.

**Supplementary Information:**

The online version contains supplementary material available at 10.1186/s13023-024-03297-5.

## Introduction

Rare disorders affect 8% of Australians, including almost 500,000 people living in Victoria, an Australian State with a population of over 6.5 million [1]. Most have diseases that begin in childhood, persist through adulthood and are inherently complex. Over a third of adult Australians living with a rare disease endure a diagnostic odyssey lasting years [2], resulting in unnecessary or delayed treatment, poorer health outcomes and substantial burden on our health system [3].

It is estimated that over 80% [4] of rare diseases have a genetic origin. Rapid evolution of high throughput sequencing approaches has dramatically increased the speed and reduced the cost of finding a genetic diagnosis. The most notable of these is exome sequencing (ES), which has become the backbone of clinical genetic testing and for some services, the first-line investigation of choice [5–7]. However, clinical ES is limited in the detection of non-coding and large copy number variants and is constrained to established gene-disease associations and well-characterised phenotypes, yielding a diagnostic rate of only 25–50% [5, 8–11]. While periodic ES-reanalysis (12–36 months) increases yield by a further 10% [12–16], for many adult patients, a diagnosis remains elusive.

Individuals with rare diseases benefit from diagnostic clarity through personalised intervention and surveillance, which improves morbidity and mortality and allows clarification of inheritance patterns and recurrence risks, enabling access to predictive and reproductive genetic testing [17]. The Undiagnosed Diseases Program (UDP) was first established by the US National Institute of Health in 2008 [18] to provide answers for individuals with rare diseases who remained undiagnosed despite exhaustive workup and to provide insight into novel disease mechanisms [19]. This evolved into the US Undiagnosed Diseases Network and subsequently into the Undiagnosed Diseases Network International [20]. This network has led to the diagnosis of thousands of individuals and advanced our knowledge of gene-disease mechanisms and genotype–phenotype correlations through extensive clinician-scientist collaboration and multi-omics integration. The International Rare Disease Research Consortium, set an ambitious goal of diagnostic genomic testing being available for almost all patients with rare diseases by the year 2020. This has not yet been achieved and further work must be done to increase implementation of genomic sequencing and analysis to achieve diagnoses [21].

Historically, UDPs have prioritised increased access to clinical genetics assessment and genomic sequencing for children with rare diseases [22], because they are deemed more likely to benefit from rapid diagnosis and treatment implementation than adults. This lack of awareness and perceived low clinical benefit for adults has limited their access to these projects, for whom a large clinical service testing gap also remains. Notably, despite evidence for utility and a comparable diagnostic yield in targetable phenotypes such as syndromic intellectual disability and multi-system disorders [23–27], very few adult-focussed UDPs exist (see Table 1).

**Table 1:** Comparison of overall diagnostic rate with other published undiagnosed disease programs

Published UDP data	Years recruiting	Site	Participants (N)	Phenotyping and Genotyping completed (N)	% Adult Participants	Phenotypes	Prior genetic testing	Main diagnostic strategy	Overall diagnostic rate (%)	Incidental findings (%)	PMID
Austin Health Adult Undiagnosed Diseases Program (AHA-UDP)	2018–2021	Victoria, Australia; monocentric	104(50 probands)	98	100%	Diverse	100%	WES reanalysis + WGS*	32	0	This paper
Program for undiagnosed rare diseases (UD-PrOZA) [28]	2015–2020	Belgium; monocentric	329	329	93.30%	Diverse	59%	WES + proteomics	18.0	7	35606766
The Italian Undiagnosed Rare Diseases Network (IURDN) [29]	2016–2019	Italian; multicentric	110	13	69.20%	Diverse	N/A	WES	53.90	0	32928283
Undiagnosed Disease Network (UDN) [30]	2015–2017	United states of America; multicentric	601	601	41.80%	Diverse	32%	WES	35.0	N/A	30304647
Spain Undiagnosed Disease Program [31]	2015–2018	Spain; multicentric	147	30	25.90%	Diverse	100%	WES	67.00	N/A	30110963
Singapore Undiagnosed Disease Program [32]	2014–2019	Singapore; multicentric	196	196	10.0%	Neurodevelopmental, congenital malformations	N/A	WES or WGS	37.20	N/A	32819910
The Korean undiagnosed diseases program (KUDP) [33]	2017–2018	Korean; multicentric	97	72	5.20%	Diverse	0%	array > NGS > exome	38.90	N/A	30894207
Initiative on Rare and Undiagnosed Disease in Japan (IRUD) [34]	2015–2020	Japan; multicentric	4205	4205	N/A	Diverse	N/A	WES	42.90	N/A	33997444
Victorian Undiagnosed Diseases Program (UDP- VIC) [22]	2016–2018	Victoria, Australia; monocentric	150	150	4%	Diverse	100%	ES > GS > RNA-seq	42.70	N/A	34740920
Finding Of Rare disease Genes FORGE) Canada Project [35]	2011–2013	Canada; multicentric	375	375	Paediatric	Diverse	N/A	WES	54	N/A	24906018
Deciphering Developmental Disorders study [36–39]	2011	UK; multicentric	13,500	13,500	N/A	Primarily neurodevelopmental	N/A	WES + array CGH	30	N/A	25529582
Care4Rare Canadian Consortium [40]	2013–2018	Canada: multicentric	830	830	Adults are recruitable	Diverse	N/A	N/A	32	N/A	36332610
Care4Rare -SOLVE [40]	2018–2021	Canada: multicentric	601	601	Adults are recruitable	Diverse	N/A	N/A	14	N/A	36332610

A comparison of the cohort characteristics and diagnostic rates between the AHA-UDP and other published UDP studies

The Austin Health Adult Undiagnosed Diseases Program (AHA-UDP) was a two-year project that aimed to address an area of unmet need for adults in Victoria with presumed orphan or undiagnosed Mendelian disorders and was designed to complement the existing UDP-Victoria, which was targeted at paediatric rare disease [22]. We used a combination of ES re-analysis, genome sequencing (GS), ddPCR, advanced bioinformatic algorithms [41, 42] and variant analysis software [43] and collaborated with local, national, and international researchers to increase diagnostic yield, expand the phenotype of known diseases, and contribute to novel gene discovery.

## Methods

### Study design and population

The AHA-UDP was a two-year project applying emerging gene discovery technologies in adult participants from a range of settings. It had two major objectives: (a) to identify the causal mutations for a variety of diseases in individuals and families enrolled over two years using the latest advances in high throughput genomic technologies; and (b) discover novel gene/phenotype relationships. It was a prospective cohort study of individuals/families identified with rare, previously undiagnosed genetic conditions.

Probands over the age of 16 years known to Austin Health were referred by their treating clinicians to the AHA-UDP team for consideration of inclusion in the program. Individuals/families were discussed at multidisciplinary case conference with subsequent recruitment offered to eligible families. The inclusion criteria were deliberately broad and included the presence, in a family, of at least one adult member who: (a) was a registered patient of Austin Health; (b) had a rare, undiagnosed condition of presumed genetic aetiology; and (c) in whom previous sequencing or microarray analysis was non diagnostic and (d) was willing to sign consent (individual or appropriate legal guardian), provide access to clinical data and to provide a DNA sample. Proposed individuals were prioritised based on uniqueness of their phenotype, the number of affected individuals within the family, individuals from different families having the same apparently novel phenotype, the presence of consanguinity, ineligibility for an alternate testing pathway or project.

Eligible families were seen through the Austin Health Genetics Service and offered enrolment. Written individual informed consent was obtained from the affected and unaffected participants in each family.

The PhenoTips [44] phenotype/genotype database was utilised. Pedigrees were created for each family and phenotypes were recorded using HPO terms. Candidate variants short listed from the bioinformatic pipeline were recorded on the database and then reviewed at the AHA-UDP Multidisciplinary team meeting. DNA samples were extracted from blood (and other tissues for the tuberous sclerosis complex (TSC) cohort) and stored using de-identified sample IDs at the Melbourne Brain Centre.

Ethical approval for the study was granted by the Austin Health Ethics Committee (HREC/18/Austin/41).

### Sequencing

The genomic analysis strategy was case-specific and based on detailed clinical phenotyping which provided diagnostic flexibility. A typical approach was the re-analysis of non-diagnostic massively parallel sequencing data (gene panel or ES) followed by genome sequencing (GS) of the proband and additional family members, as appropriate.

Trio GS (Novogene—Illumina NGS) was performed for an affected proband and their unaffected parents where a de novo mutation or autosomal recessive (AR) pattern of inheritance was suspected. Trio/Quad GS was also performed as appropriate where there were multiple affected individuals in a family (sequencing of more than one affected individual increases the diagnostic yield). In other individuals singleton GS was undertaken (where GS of parents and/or other affected individuals was either not practicable or not thought likely to significantly increase yield).

Variants were reported using the GRCh38/hg38 reference genome. SNP and indel variants were called using GATK v4.1.9.0 [45, 46]. Structural variants were called using an ensemble of methods; CNVnator v0.4.1 [47], Manta v1.6.0 [48] and smoove v0.2.5 [49].

Variant filtering was performed using in house pipeline Cavalier and SVPV v1.00 [43], based on annotation by Ensembl VEP v104.3 and subsequent filtering by variant impact, affected genes and allele frequency in gnomAD. Variants were visualised using IGV snapshots and SVPV pdf generated for candidate variants using Cavalier, an in-house method (available from https://github.com/bahlolab/nf-cavalier). Variants were classified based on review of Cavalier presentation slides.

The following tools were used to examine genomic sequencing data for expansions: exSTRa v0.9.0.0 (expanded STR algorithm: detecting expansion with paired-end Illumina sequencing data [42]), ExpansionHunter v3 (algorithm for expanded known STR detection[50]), and ExpansionHunter Denovo v0.9.0 (genome wide novel STR detection algorithm[41]).

Candidate variants in genes without known disease associations were uploaded to the GeneMatcher database with patient consent.

### Microarray analysis

Microarrays were performed on a subset of samples using the Illumina Infinium Omni 2.5 SNP microarray platform.

### TSC1/2 mosaicism analysis

Targeted gene sequencing of coding regions and splice sites was performed on DNA extracted from blood and other affected tissues (see table [51]). Libraries were prepared and enriched using Sureselect XR target enrichment (Agilent Design ID 0825941). Indexed libraries were pooled and sequenced to a targeted coverage of 700–1000 reads/base (Illumina NextSeq500, 2*75 bp). Seqliner v0.8 was used to generate aligned reads and call variants against the hg19/GRCh37 human reference genome. PathOS v1.5 was used to annotate and transform variants to standard nomenclature and filter for rare, non-synonymous variants within 20 bp of coding exons. Copy number was analysed using Gaffa 3.0 Targeted. Data was analysed on CLC Genomics Workbench v12.0.2 to screen for low frequency variants present at > 1% VAF. Mosaic variants were then validated with ddPCR as described previously [51].

### Report generation and return of results

Following analysis, each case was discussed in a MDT teleconference comprising clinical geneticists, genetic counsellors, clinical scientists and bioinformaticians. Reports were written collaboratively using a cloud office platform before signing off by PI. Participants were notified of their results on completion of the study by letter (referring clinician cc’d) and offer of a follow up appointment, unless a variant of potential clinical significance was found with possible reproductive implications, in which case they were notified sooner. Validation of research results by the referring clinician using a NATA accredited clinical laboratory was recommended.

Mirroring UDP-Vic [22], families were considered solved when a molecular diagnosis in an established disease gene was made, or when a strong candidate for novel gene discovery was identified. For molecular diagnoses in established disease genes, the variant needed to reach criteria for the American College of Medical Genetics and Genomics (ACMG) classification of pathogenic or likely pathogenic (P/LP) [52]. For novel disease-gene variants and new phenotypes in known disease genes, we adopted the same ‘strong candidate diagnosis’ approach as UDP-Vic [22]. This required the candidate diagnosis meet the following three criteria: (i) phenotypically similar unrelated individuals (matched through data sharing platforms) with a variant in the same gene and population allele frequencies compatible with disease penetrance and inheritance pattern; (ii) in vitro or in vivo functional validation, either planned or underway via local or external research collaborators; and (iii) multidisciplinary agreement that variants in the proposed gene(s) were likely causative for the phenotype(s) and recommended for functional confirmation.

Families were listed as having a variant of uncertain significance (VUS) when they were unsolved, and when: (a) a plausible variant in known gene was found but that variant did not meet Pathogenic/Likely Pathogenic ACMG criteria, or (b) a potential novel disease-gene or known disease gene with a novel phenotype was found, but there was insufficient evidence for the variant to be considered a strong candidate.

## Results

### Participants

The AHA-UDP commenced in December 2018 and recruited the final family in March 2021. Most cases were referred by a Clinical Geneticist or Neurologist for consideration to the AHA-UDP team. One hundred and fourteen individual participants from 51 families were offered enrolment in the project; one individual withdrew from the study and one individual did not provide a sample, resulting in 104 participants from 50 families who participated in the study.

All probands were adults at time of enrolment, although many had symptom onset in childhood. Four affected relatives under the age of 16 were recruited for potential variant segregation. In the 50 families who participated, the primary phenotype (based on HPO terminology) of the condition under investigation included an abnormality of the nervous system (HP:0000707) (n = 30), a risk of neoplasm (HP:0002664) (n = 12), an abnormality of the genitourinary system (HP:0000119) (n = 4), an abnormality of the cardiovascular system (HP:0001626) (n = 2), an abnormality of the endocrine system (HP:0000818) (n = 1), an abnormality of metabolism/homeostasis (HP:0001939) (n = 1) and an abnormality of the musculoskeletal system (HP:0033127) (n = 1). Eight of the twelve families with a condition associated with a risk of neoplasm were recruited because they were suspected to have mosaic TSC. In those with an abnormality of the nervous system, there were six who were recruited because the proband had intellectual disability believed to be due to an undiagnosed syndrome. Table 1 shows a comparison of the AHA-UDP cohort and diagnostic rates with other published UDP studies. Table 2 shows the characteristics of the AHA-UDP cohort. Table 3 shows a summary of the genetic testing undertaken in the cohort. See supplement 1 for a clinical summary of all families. See supplement 2 for tabulated results and outcomes for all families.

**Table 2:** Summary of AHA-UDP cohort

Participants	
Total number of participants*	104 (50 probands)
Sex	55M, 57F
Age at enrolment of participants tested (mean)	48 (SD = 17, 95% CI 44–52)
Total number of clinically affected individuals	73
Total number of families	50
Number of families with a single affected individual	38
Clinical phenotype^	
Abnormality of the nervous system (HP:0000707)	30 (60%)
Intellectual disability believed due to an undiagnosed syndrome	6 (12%)
Risk of neoplasm (HP:0002664)	12 (24%)
Tuberous Sclerosis Complex (TSC)	8 (16%)
Abnormality of the genitourinary system (HP:0000119)	4 (8%)
Abnormality of the endocrine system (HP:0000818)	1 (2%)
Abnormality of the cardiovascular system (HP:0001626)	2 (4%)
Abnormality of the musculoskeletal system (HP:0033127)	1 (2%)
Abnormality of metabolism/homeostasis (HP:0001939)	1 (2%)
Tests prior to enrolment	
Karyotype	3
Chromosome microarray	26
Fragile X	6
Methylation testing	3
Single gene/s testing	17
Gene panel	11
Singleton ES	25
Trio ES	3
ES reanalysis	1

The characteristics of the AHA-UDP cohort. *Some participants were unaffected family members enrolled for trio GS or exclusion mapping. ^The primary phenotype (based on HPO terminology) of the condition under investigation

**Table 3:** Summary of testing and outcomes

Testing through UDP (per family)	
High resolution array	5
Single gene/s testing	11
Gene panel re-analysis	1
Singleton whole exome sequencing	1
Exome reanalysis	14
Singleton whole genome sequencing	21
Trio whole exome sequencing	1
Trio whole genome sequencing	8
Quad whole genome sequencing	3
Short tandem repeat analysis	4
ddPCR validation	10
Functional studies	3
Main outcomes	
Solved	16 (32%)
Candidate VUS	18 (36%)
No candidate	16 (32%)

A summary of the tests arranged and main outcomes of the study

### Diagnoses

**Fig. 1:**
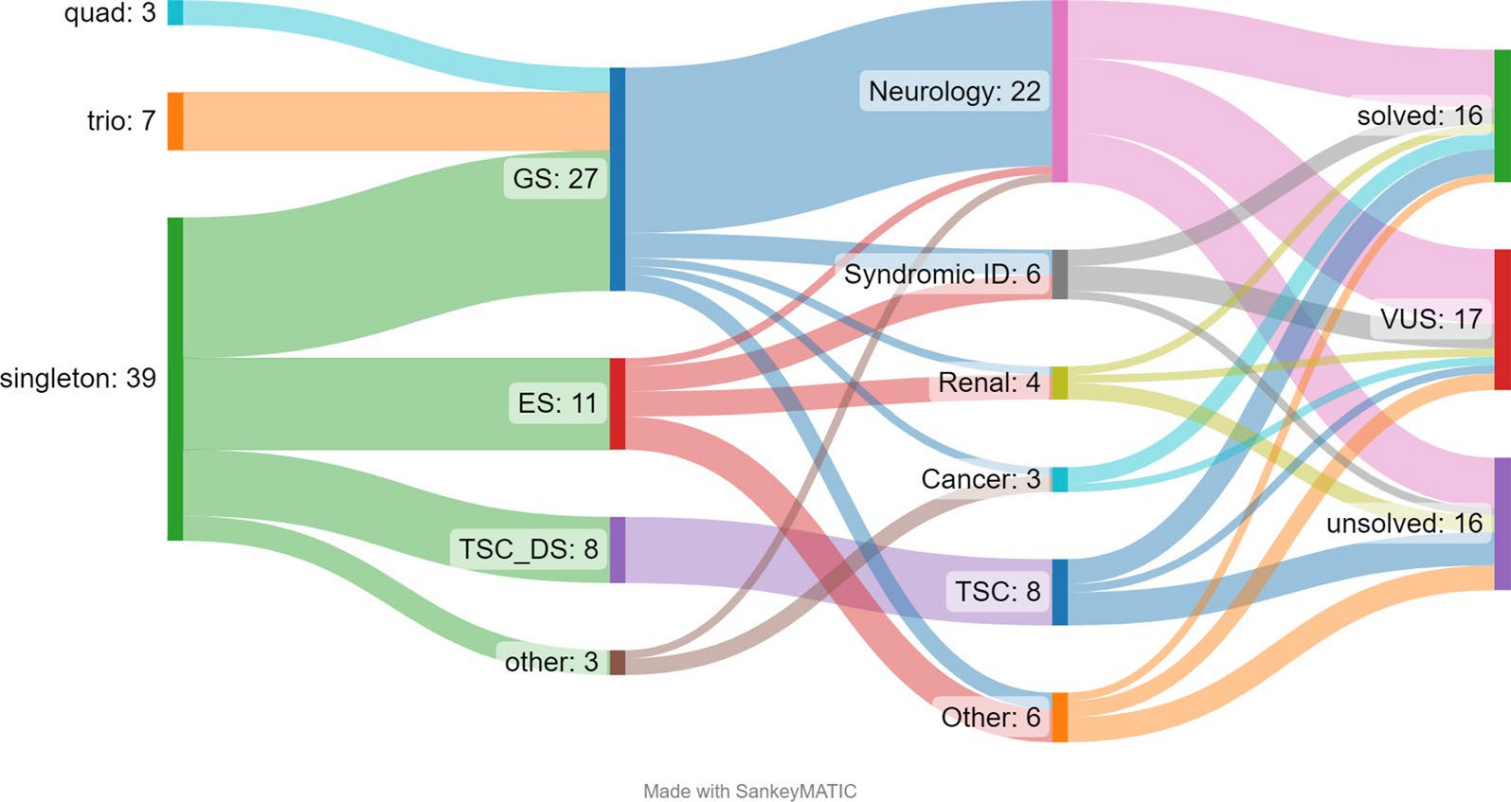
Sankey diagram of AHA-UDP analysis

Ninety-eight individuals from the 50 families were phenotyped and genotyped. All probands had standard clinical genetic testing appropriate to their phenotype prior to recruitment. Singleton GS was completed for 21, trio/quad GS analysis was completed for 11, ES reanalysis was completed for 14, and TSC1/2 deep sequencing (700-1000x) to detect low level mosaicism was undertaken for 8. This was followed by ddPCR validation at Melbourne Brain Centre for those found to have a candidate mosaic variant. Validated mosaic *TSC1* or *TSC2* candidate variants were identified in 4/8 individuals, *PTEN* analysis via the Peter MacCallum Cancer Centre laboratory was undertaken for one patient, and *PRKACB*, *TOP3B* and *NARS* functional studies were undertaken in three respective families. Chromosomal microarray was undertaken in multiple tissues and individuals in one family. Confirmatory microarray was undertaken in one family. In total, 16/50 families had a diagnosis or strong candidate diagnosis made (32%). One or more candidate variants of uncertain significance were detected in a further 18/50 families (36%). No candidate variants were identified in 16/50 families (32%) (see Fig. 1). Table 3 contains a summary of the tests arranged and main outcomes.

### Published case demonstrating a novel method for rapid short tandem repeat detection

#### NOP56/SCA36 (AH006)

Spinocerebellar ataxias are often caused by expansions in short tandem repeats (STR). Recent methodological advances have made repeat expansion (RE) detection via GS feasible. We published a family demonstrating the use of the ExpansionHunter and exSTRA algorithms to provide a clear and rapid diagnosis of *NOP56* SCA36 due to the intronic GGCCTG motif expansion in three affected individuals in a single family with a clinical diagnosis of SCA [53]. A diagnosis was obtained within five days of receiving the sequencing data. A clinical examination confirmed symptoms consistent with SCA36. Molecular diagnostics for SCA36 are not available in Australia- testing was sought via two international providers (Fulgent, Temple City, CA, and MNG Laboratories, Atlanta, GA). Both returned a positive result showing an expansion of a single allele > 70 repeats, however neither diagnostic lab offers exact size estimation methods (such as Southern blot) for this locus.

### Novel disease gene discoveries

#### TOP3B (AH035)

A novel homozygous deletion was found that included *TOP3B* was found on an Infinium Omni2.5 (Illumina) microarray in an individual with bilateral renal cancer [54]. Topoisomerase III beta is one of the least understood members of the topoisomerase family of proteins. Immunoblotting with an antibody against TOP3B showed no detectable protein. Analysis in both the patient and modelled human cells showed that disruption of *TOP3B* causes genome instability with a rise in DNA damage and chromosome bridging (mis-segregation due to formation of Halliday junctions). The molecular defect underlying this pathology is a significant rise in R loop formation (post processing mRNA/DNA dimers). This identified *TOP3B* as a putative cancer gene and supports recent data that R loops are involved in cancer aetiology.

#### PRKACB (AH033)

We identified a de novo missense variant in *PRKACB* on ES re-analysis in an individual with ID, refractory focal epilepsy, spasticity, periventricular nodular heterotopia, a common atrium / AVSD, polydactyly and several tumours (benign ovarian tumour, liver haemangioma and renal cell carcinoma). *PRKACA* and *PRKACB* encode two catalytic subunits of cAMP dependent protein kinase (PKA). This case was reported along with 3 other *PRKACB* variants and 3 affected individuals with the same *PRKACA* variant (one family) [55]. All affected individuals had the same novel phenotype of atrioventricular septal defects or a common atrium, along with postaxial polydactyly and other features including skeletal anomalies, ectodermal defects of variable severity and cognitive deficits.

### Novel disease gene phenotype relationship discoveries

#### NARS (AH025)

In collaboration with the NARS project at University of Antwerp [56] a missense variant in the NARS protein was identified [c.1025G > A (p.Cys342Tyr)]. Two affected children (LD, DD) had an onset of neuropathy symptoms at age 18 and 19 respectively. Relatively rapid progression of bilateral lower limbs more than upper limbs weakness over 6 months was noted, with subsequent sensory involvement. NCS identified sensori-motor neuropathy with low normal conduction velocities. Their father had small fibre neuropathy symptoms (burning pain in the feet) with onset age 55 whilst the mother was asymptomatic.

#### KMT2C (AH016)

A 200 kb intragenic heterozygous microdeletion (exons 2–43 of 59) was detected in the *KMT2C* gene on microarray analysis in a proband with mild ID and renal cancer. Loss of function variants in *KMT2C* have been reported in association with a variety of neurodevelopmental phenotypes; autism, a Kleefstra syndrome-like developmental phenotype [57–60], schizophrenia susceptibility, bipolar disorder, and cancer predisposition. Li et al. [59] described a germline mutation in *KMT2C* (MLL3) in four individuals from a multigenerational Chinese family with colorectal cancers and acute myeloid leukemia. Sasaki et al. [60] reported three different variants in *KMT2C* (MLL3) in three closely related family members with familial nasopharyngeal carcinoma using exome sequencing. One variant [p.(Tyr816*)] was predicted to result in a premature stop codon and loss-of-function, the other two variants were missense variants. They hypothesised that inactivating mutations of *MLL3* may be associated with a highly penetrant and previously unknown cancer-predisposition syndrome. No functional studies were performed (PMID: 26014803). Somatic variants in the *KMT2C* gene are often identified in renal cell cancer [61]; however, none of the families reported with germline alterations in this gene have had renal cancer. This variant is classified as pathogenic according to the ACMG criteria[52] (PVS1, PM2, PS2).

There is therefore sufficient evidence to conclude that this microdeletion explains the individual’s ID and there is limited evidence to suggest that haploinsufficiency of *KMT2C* is cancer predisposing.

### Mosaic TSC

Eight individuals with a definite clinical TSC diagnosis or suspected TSC were enrolled in the AHA-UDP project (see supplement 3). All had prior negative standard of care NGS TSC sequencing. Approximately two thirds of TSC is de novo [62] with mosaicism known to be common in this group and likely accounting for the relatively low sensitivity (~ 85%) of conventional Sanger or NGS sequencing for individuals with a clinical diagnosis of TSC [62]. Therefore, a strategy of deep sequencing (700–1000* coverage) and ddPCR validation of low level mosaic variants was adopted [51]. In three individuals mosaic low level variant allele fraction (VAF) strong candidate *TSC2* variants were identified and validated. In a further individual a mosaic low level VAF candidate variant was identified but failed ddPCR validation. In another individual a mosaic *TSC1* VUS was identified. The AHA-UDP TSC cohort has been published as part of a collaboration between our groups and a Chinese paediatric TSC cohort [51]. These results are in keeping with previously published cohorts of TSC patients showing a high level of *TSC1* or *TSC2* pathogenic variant mosaicism in individuals with a definite or suspected clinical diagnosis of TSC, in particular for *TSC2* variants, which account for 92% of mosaic patients [63]. This is in contrast to germline TSC where *TSC2* accounts for 69% of cases [64–70]. Of people with a previous NVI (no variant identified) after conventional TSC testing it is estimated that ~ 50% are mosaic [51, 63].

## Discussion

### Case selection mirrors paediatric UDP cohorts

This study identified a cause for 32% of individuals/families recruited to an adult UDP program with a further 36% having a genomic finding that did not meet criteria for a definite diagnosis.

We placed an emphasis on recruiting patients with neurological conditions—there are approximately 7211 (OMIM) [71] phenotypes with a known genetic molecular basis; caused by pathogenic variants in approximately 4660 genes. Panel App Australia [72] lists 3935 green genes on their “Mendeliome” gene panel (v1.1894) and 5382 genes in total (including putative disease genes). Of these phenotypes a significant proportion are neurological. For example for intellectual disability syndromes (v0.6063) alone there are 1621 known causative genes (Panel App Australia) [72]. A subset of recruits were judged to have phenotypes likely due to repeat disorders. Molecular diagnosis for dominant ataxia repeat expansions (SCA 1, 2, 3, 6 and 7) are readily available, however no molecular diagnostics are available in Australia for rarer ataxias such as SCA36, or recently discovered ataxias such as CANVAS [73] and SCA27B [74]. The rapid diagnosis of SCA36 highlights the utility for WGS in the diagnosis of rare repeat expansion disorders.

### Diagnostic rates are equivalent

Paediatric UDP program diagnostic rates are generally higher than comparable adult cohorts [23] due to differences in case selection (severe neurodevelopmental phenotypes and/or congenital malformations) and low survivability of the most severe disorders into adulthood. Additionally, patients with the more severe genetic conditions may already have been diagnosed in childhood. It is of note that prenatal diagnostic rates are higher again than similar paediatric cohorts [75], likely for similar reasons. Also of note is that rare diseases are a leading cause of death in childhood whereas this is not the case in adults (only ~ 5% of adults have a rare genetic disease) [4]. An Italian population based study [76] compared the mortality rates across several types of rare genetic diseases in adults and demonstrated improved efficacy of treatment and reduced mortality through genetic diagnosis. The diagnostic rate from the only other adult-focused UDP identified in the literature (UD-PrOZA) was 18%.

In adult UDPs (AHA-UDP and UD-PrOZA) with careful case selection, and controlling for severity (survivability), the diagnostic rates were equivalent to paediatric cohorts; children with undiagnosed rare diseases become adults, and the population prevalence of genetic conditions are unlikely to change significantly over time (although, the proportion with a diagnosis will increase). Lower diagnostic rates could also be explained by a loss of history or medical information over time with deep phenotyping often being key to diagnosis, as well as less capacity for family (trio) sequencing if relatives are deceased or unavailable. Adults are also likely to have other medical phenotypes in addition to those caused by their genetic condition, further complicating diagnosis. Adult-onset multifactorial diseases are often difficult to distinguish from less common monogenic diseases with similar phenotypes (e.g. familial cancer syndromes, maturity onset diabetes of the young (MODY), familial hypercholesterolaemia (FH), cardiomyopathy syndromes, dementia syndromes etc.). This makes case selection more challenging. It is generally considered that a > 10% chance of a monogenic condition is an acceptable threshold for the deployment of genetic testing in adults. This figure comes from cancer genetics [77], is to some extent arbitrary, and is dependent on the cost of genetic testing at a given time. Additionally, trio analysis is less likely to yield additional diagnoses in adults (even when parents are available), because a greater proportion of adult-onset genetic disease follows an autosomal dominant pattern of inheritance and a smaller proportion of adult genetic disease is due to de novo mutations in the proband, rendering the trio de novo approach less powerful.

The AHA-UDP project met its aims of demonstrating the utility of novel analysis techniques (exome re-analysis, genome sequencing, deep sequencing, ddPCR, novel short tandem repeat detection algorithms) in improving diagnostic rates and identifying novel gene disease associations. We identified a diagnosis in 32% of the enrolled families (all of whom had had uninformative previous standard of care clinical diagnostic genetic testing). We identified two novel disease genes and five new genotype–phenotype associations. The detection of a rare repeat expansion in one family highlights the exceptional utility of these novel repeat expansion analyses; particularly in adult disease programs given that many of the diseases caused by repeat expansions have onset in adulthood.

Our diagnostic rates are potentially subject to ascertainment bias as most referrals were from Clinical Geneticists or experienced Neurologists, and cases were selected on the basis of a perceived high prior probability of a monogenic cause. However, genomic testing is still at a premium and so it is likely that in most clinical and research contexts where it is being utilised as a diagnostic test that it would be reserved for cases judged to have a reasonable diagnostic yield.

A study of the illness narratives of participants in the American UDN [78] compared the experiences of adult probands with those of parents of paediatric probands. They found that adult probands hoped a diagnosis would help them resume their former lives. In contrast the parental group understood that their child was unlikely to be cured but hoped a diagnosis would lead to an improvement in medical management. For adult patients, frustration often stemmed from the need to validate symptoms in light of nondiagnostic testing, while for parents it was caused by a concern that evidence crucial to making a diagnosis was being overlooked. That adult patients and parents experience undiagnosed conditions differently, demonstrates the need to further understand and address the psychological impact of undiagnosed conditions in these groups in order to provide optimal support.

Federally funded genomic testing through the Medicare program to identify the cause of childhood syndromes and intellectual disability for children aged ten years and younger became available in Australia on 1 May 2020 [79] due to the evidence that had been gathered from paediatric populations[80]. However, for individuals in Australia with an undiagnosed syndrome who are older than 10 years of age, publicly funded diagnostic testing remains difficult to access because the evidence for utility in adulthood has been underexplored. Yet clear cases for adult-onset genetic testing already exist for example for conditions where disease prevention is possible through screening, risk reducing surgery or chemoprophylaxis and for conditions associated with severe outcomes where predictive testing can enable future planning for at risk individuals.

### There can be unique benefit to adults of reproductive age

By way of example, we targeted mosaicism and milder conditions that do not present in childhood. Through targeting mosaicism in patients with suspected TSC and previous uninformative conventional TSC sequencing, we were able to confirm mosaic likely disease causing mutations in 3 out of 8 individuals analysed and a mosaic variant of uncertain significance was identified in one further individual. This is consistent with other recent reports that mosaicism is more common in TSC than had previously been recognised.

### Adults with undiagnosed conditions are generally underserved

As the utility of UDP programs becomes more established they will leverage novel technologies to increase diagnostic yield. The Austin UDP group will be involved in recruiting patients (initially re-analysing data) to the Undiagnosed Diseases Network-Australia (UDN-Aus) [81]. This is an Australia wide project which began recruiting patients in 2022, leveraging a partnership with the Broad Institute [82] and their *Seqr* platform [83] to enable cloud-based exome/genome analysis by the recruiting clinician (rather than a bioinformatician). The intention is that this model will increase access to the UDP model for clinicians and patients and enable upskilling of clinicians in genomic analysis and a more cost effective/ sustainable model for UDP delivery.

The recruitment and diagnostic framework established by AHA-UDP will continue to be utilised to identify, consent and phenotype adult patients with likely monogenic undiagnosed diseases. Suitable cases will be submitted for consideration of recruitment to the recently activated nationwide Australian Undiagnosed Diseases Network (UDN-Aus).

## Conclusion

Here we describe the outcomes from AHA-UDP. We hope our results will encourage other, similarly placed hospitals to also embark on such programs.

The AHA UDP study was conceived to address the absence of a UDP project for adult patients with rare/monogenic disease in Australia. The study met its aims in terms of demonstrating the utility of an adult UDP framework in achieving comparable diagnostic rates to previously published paediatric UDP studies and in addition a comparable rate of novel genotype phenotype relationship discovery. This has been demonstrated by the results presented in this paper and the already published papers arising out of this study.

### Supplementary Information

Additional file 1.

Additional file 2.

Additional file 3.

## Data Availability

Sequence data is stored at the Walter and Eliza Hall Institute of Medical Research. Supplemental resources are available online. Additional information not subject to ethical restrictions can be obtained from the corresponding author upon request.
