# Supplementary material for: Experience of the first adult-focussed undiagnosed disease program in Australia (AHA-UDP): solving rare and puzzling genetic disorders is ageless
Source: Orphanet J Rare Dis. 2024 Aug 2;19:288. doi: 10.1186/s13023-024-03297-5 (PMC11297648; doi:10.1186/s13023-024-03297-5)
Supplement: Supplementary file 1 — Additional file 1. [file 13023_2024_3297_MOESM1_ESM.docx]

# **Appendix 1 - Summary of solved cases**

AH002: Proband 48M: An in-frame deletion of exons 4-7 of the *SPAST* gene was identified on singleton GS for an Individual with a personal and family history of hereditary spastic paraparesis (HSP). This was a 26.7 kbp deletion which could not have been picked up on microarray. This variant is consistent with a diagnosis of ADHSP type 4. MIM#182601

AH006: A *NOP56* STR motif repeated at least 800 times was detected on trio GS (3 affected individuals; 64M,70M,89F) in an individual with ataxia and family history of AD hereditary ataxia affected 9 individuals. This variant is consistent with a diagnosis of SCA36.(1)

AH016: Proband 52M: an intragenic microdeletion of exons 2-43 of *KMT2C* was detected on microarray analysis in an individual with a history of mild ID and renal cancer. This was confirmed using ddPCR from a separate blood sample and was detected in only 2/6 renal cancer samples using ddPCR because some tumour samples were of poor quality. The microdeletion was found to be mosaic in the proband’s father. Loss of heterozygosity was not detected on ddPCR in any of the renal cancer DNA samples. This result is consistent with a diagnosis of Kleefstra like syndrome in the proband. *KMT2C* is a histone methyltransferase which regulates gene transcription by remodelling chromatin. It has been hypothesised that heterozygous loss of function variants in *KMT2C* are associated with an increased lifetime risk of certain cancers. However, if this is the case it is not currently clear if this is associated with a second somatic hit in the gene in cancer tissue. ACMG criteria; Pathogenic - PVS1 - null variant, PM2 - absent in population, PS2 - de novo.

AH018: Proband 36M. A previously reported pathogenic missense variant c.55C>T (p.(Arg19Trp)) was identified in the *SPTAN1* gene on singleton GS. Pathogenic variants in *SPTAN1* have been reported in association with autosomal dominant hereditary spastic paraplegia +/- cerebellar ataxia, hereditary motor neuropathy, sensorimotor neuropathy and developmental disorder, epilepsy, autism and intellectual disability. The p.(Arg19Trp) variant has previously been reported in multiple (seven) patients with HSP. ACMG classification; Pathogenic - PP1-S -co segregation with disease, PS4 - absent in population, PS4_M - cases>controls.

AH021: Proband 19F The variant c.1714_1716+4dup was detected in the *TSC2* gene on deep NGS sequencing and validated with ddPCR at ~1.9% Variant Allele Frequency (VAF). In a patient with suspected mosaic TSC (facial angiofibromas, renal AML). This duplication of 7 bases, involving the canonical splice site in intron 16 is predicted by in silico splicing prediction programs (MaxEnt(2), NNSPLICE(3) and SSF(4)) to likely disrupt the canonical donor splice site and generate a cryptic splice site. This variant was confirmed using RT-PCR of RNA from the patient to disrupt splicing of *TSC2* by causing exon 23 skipping(5). This variant has not been reported in ClinVar(6) or LOVD(7) databases, or to our knowledge, in the literature. This variant has not been observed in population databases (ESP(8) and gnomAD(9)). ACMG classification - VUS - PM2 -Absent from controls, PP3 - Multiple in silicos predict likely deleterious (MaxEnt, NNSPLICE, SSF).

AH025: Probands 34M, 39F *A* personal and family history of Charcot Marie Tooth (CMT). Two affected siblings with onset of neuropathy symptoms at age 18 and 19 respectively. There was relatively rapid progression of bilateral lower limbs more so than the upper limbs weakness over 6 months, with subsequent sensory involvement. Nerve conduction studies revealed a sensorimotor neuropathy with low normal conduction velocities in both. Their father had small fibre neuropathy symptoms (burning pain in the feet) onset age 55, whilst their mother was asymptomatic. A missense variant- c.1025G>A (p.Cys342Tyr) in *NARS* was identified in the affected family members. *NARS* has recently been linked with a novel inherited peripheral neuropathy (unpublished cohort). This family is part of an ongoing research collaboration with the *NARS* project at University of Antwerp for functional studies. Pathogenic variants in *NARS* have previously been known to be associated with AR/AD Neurodevelopmental disorder with microcephaly, impaired language and gait abnormalities (MIM#619091, 619092). ACMG classification - VUS - PM2 - Absent from population databases, BP4 - Multiple in silicos predict benign effect (SIFT, Polyphen 2).

AH026: Proband 35F. A c.4357G>T (p.(Glu1513*)) variant was detected in the *TSC2* gene on deep NGS sequencing and validated with ddPCR with a 2%MAF, in a patient with suspected mosaic TSC due to multiple renal angiomyolipomas and subependymal giant cell astrocytoma. The p.(Glu1513*) variant has been previously reported as pathogenic in two patients with tuberous sclerosis(10,11). ACMG classification: Pathogenic - PVS1 - Null variant, PM2 - absent in population, PS4-M Cases>Controls.

AH027: Proband 51F. Heterozygous missense variants in WFS1 (c.728C>T; p.(Ala243Val) and c.400G>A; p.(Ala134Thr)) were identified on singleton GS in a patient with progressive myoclonic ataxia, mild ID, asthma, type 2 diabetes mellitus (DM), schizophrenia, depression, anxiety, panic attacks, hearing loss and obesity. Pathogenic variants in WFS1 are associated with autosomal recessive Wolfram syndrome spectrum disorder (MIM#222300); this is a progressive neurodegenerative disorder associated with; DM, optic atrophy (absent in this patient), other endocrine abnormalities, sensorineural hearing loss, progressive neurological abnormalities (cerebellar ataxia, peripheral neuropathy, dementia, psychiatric illness, and urinary tract atony). ACMG criteria - c.728C>T - VUS - conflicting in silicos, SIFT tolerated, Polyphen2 possibly damaging, Grantham 64, Gnomad MAF 0.0002984; c.400G>A - VUS - conflicting in silicos, SIFT tolerated, Polyphen 2 possibly damaging, Grantham 58, gnomAD MAF 0.0001368

AH028: Proband 25F. Singleton ES re-analysis found a maternally inherited *PHIP* missense variant (p.(Phe277Tyr)). Chung-Jansen syndrome was suspected (mother also suspected to be affected). This individual had a Rett-like phenotype with severe ID, impaired mobility, non-progressive thoracolumbar scoliosis and hip subluxation. The head circumference was between 3rd and 15th centile, and this individual has hand automatisms and lives with a carer. ACMG criteria; this PHIP variant was absent from population databases (PM2) and in silicos were conflicting, CADD score was 23.7 (>20 predicts deleterious), SIFT predicted damaging and Polyphen 2 predicted benign.

Exome sequencing from an external laboratory (Perkin Elmer) identified- VUSs in *ARID2*, *CAD*, *CHD2* and *TRIO*. Chromosomal microarray (CMA) identified a copy number change of unknown significance; a duplication of region 10q24.1 [GRCh37 chr10:98287792-98716810 (x3)] as well as two small regions of long contiguous stretches of homozygosity (LCSH) [chr11:27781979-33195261; chrX:81677224-83678300]. Normal G-banded karyotype, normal Fragile X testing, normal *MECP2* sequence, Angelman syndrome/Prader-Willi syndrome methylation testing, urine metabolic screen, plasma amino acids, and serum transferrin isoforms.

AH031: Proband 63F. A deletion of exons 18-22 of *PKD1* was detected on GS analysis in an individual with clinical ADPKD and a FH of 13 individuals with ADPKD.

AH033: Proband 41F A de novo missense variant in *PRKACB* (c.262C>A; p.(His88Asn)) was detected by trio ES in an individual with ID and epilepsy with congenital heart disease (atrioventricular septal defect/common atrium) and polydactyly. *PRKACB* encodes one of the two catalytic subunits (Cβ) of cAMP-dependent protein kinase (PKA). De novo missense variants in *PRKACB* were recently reported in four unrelated individuals (including our patient) to cause a multiple congenital malformation syndrome(12) known as cardioacrofacial dysplasia type 2 (CAFD2). The four variants in these patients were all de novo and absent in the gnomAD database; Investigation of the functional consequences of the variants found that they lead to PKA holoenzymes which are more sensitive to activation by cAMP than the wild-type proteins, and these enzymes lead to inhibition of hedgehog signalling(12). Shared phenotypic features include congenital cardiac defects, primarily common atrium, or atrioventricular septal defect; limb anomalies, primarily postaxial polydactyly; and dysmorphic facial features. Two of the three other patients with *PRKACB* variants had mild ID/developmental delay with other neurological features. Unusual tumours were not reported in any of the other patients. ACMG classification; *PRKACB* - Pathogenic - PS2 (de novo), PM1 - known functional domain (G-Loop), PS3 - Established functional studies.PP4 - gene highly specific for phenotype. PIGR - VUS - PS2- (de novo).

AH035: Proband 56M. A homozygous deletion including the whole of the *TOP3B* gene was detected by microarray in an individual with bilateral renal cancer and cytogenetic evidence of instability. This patient was reported by Zhang et al(13) who found *in vivo* evidence that *TOP3B* loss leads to increased R-loop formation. Their hypothesis was that loss of *TOP3B* causes increased the formation of R-loops, resulting in unresolved recombination intermediates that persist into mitosis leading to genome instability. Loss of *TOP3B* would therefore be expected to increase DNA damage and genomic instability.

AH038: Proband 46F. A missense variant c.388G>A (p.(Gly130Arg)) in *EIF2AK2* was identified on singleton ES reanalysis in an individual with AD dystonia. Pathogenic variants in this gene are associated with AD/AR Dystonia Type 33 (MIM#619687).(14–16) This causes a progressive focal or generalised dystonia presenting in the first decades of life. This particular variant has previously been reported in 2 families as a recurrent missense variant segregating with this disease(14,16). This variant is absent from population databases. Functional studies have demonstrated a deleterious effect on protein function. ACMG criteria; likely pathogenic; PM2-absent from controls, PS3-Functional assay, PP1-Mod - segregating with disease (? how many meioses),- PS4 - supporting (cases>controls), PS3 - supporting - functional studies show a deleterious effect.

AH039: Proband 45M. A nonsense variant **(**c.1168C>T; p.(Arg390*)) was identified on singleton GS in the *AFG3L2* gene in a patient with severe, progressive cognitive decline, ataxia and other neurological symptoms (SCA28)(17,18). Pathogenic variants in this gene are associated with an autosomal dominant form of spinocerebellar ataxia. ACMG classification; Pathogenic - PVS1 - null variant, PP4 - gene specific to phenotype, PM2 - absent in population.

AH045: Proband 73M A missense variant (c.2749G>A**;** p.(Asp917Asn)) was identified on singleton GS in the *FAT2* gene in an individual with a personal and family history of cerebellar ataxia. Pathogenic variants in this gene have recently been reported to cause an autosomal dominant cerebellar ataxia syndrome (SCA45)(19) . ACMG criteria; VUS - PM2 - absent from population databases, PP3 - Multiple in silicos predict deleterious (SIFT deleterious, Polyphen2 probably damaging).

AH046: Proband 32F an individual with a likely mosaic form of TSC (Renal AML, cortical tubers, seizures, LAMs), had high depth NGS sequencing of the *TSC1* and *TSC2* genes was previously reported negative by a diagnostic laboratory, using DNA derived from lymphocytes. Reanalysis of this data identified a mosaic *TSC2* variant below the VAF level of detection for the diagnostic lab calling (4.3%), and this variant was subsequently validated via ddPCR in several other tissues (saliva, fibroblasts, buccal, urine) (Supplemental materials 3). Prior to this finding, the female individual conceived a child via egg donation due to diagnostic uncertainty and the risk of non-mosaic TSC in an offspring. Molecular confirmation of mosaic TSC in this patient led to reproductive confidence and the patient went on to conceive naturally, with a plan for postnatal genetic diagnosis.

# **Appendix 2 - Summary of cases with candidate variants identified**

AH001: Proband 47F: An 11bp deletion l in the *BSN* gene was identified on singleton GS in an individual with Gomez-Lopez-Hernandez syndrome/Rhombencephalosynapsis. There is no known human phenotype associated with variants in this gene.

AH004: Proband 23M: A missense variant was identified in the *SYP* gene and compound heterozygous variants were identified on trio GS in the *SLITRK5* gene in an individual with syndromic severe ID, bilateral orchidopexy, alternating esotropia with nystagmus, nephrocalcinosis and hypercalciuria, bilateral adducted thumbs requiring surgery. An MRI brain was normal. Pathogenic variants in *SYP* cause X-linked recessive non syndromic intellectual disability +/- epilepsy. *SLITRK5* is not known to be linked to human disease. ACMG criteria; SYP - VUS- PS2 - de novo, PP3 - in silicos predict deleterious, *SLITRK5* del - VUS - No relevant criteria, *SLITRK5* missense - VUS - No relevant criteria.

AH005: Proband 34F: A missense de novo variant in the *PRPH2* gene was identified on trio GS in an individual with paroxysmal nocturnal haemoglobinuria, progressive weakness, seizures and dystonia. Pathogenic variants in *PRPH2* are associated with retinal diseases. No known link with patient’s phenotype.

AH007: Proband 47M: A variant was identified on singleton GS in a splice acceptor site of the *PNPT1* gene for a patient with child onset ataxia with 9 affected family members. This variant is in a region in linkage disequilibrium with the affected family members. However, *PNPT1* is associated with an autosomal recessive mitochondrial disorder called combined oxidative phosphorylation deficiency 13. This is not a good phenotypic fit for this family and the condition appears to be following an AD pattern of inheritance in the family.

AH008: Proband 49M. Missense variants were identified on singleton GS (and ES re-analysis) in *SYTL5* and *PAX8* in an individual with hypogonadotropic hypogonadism. No known human disease phenotype linked to *SYTL5* but potentially interesting gene for future research as it has pituitary expression. Pathogenic variants in *PAX8-* are associated with autosomal dominant hypothyroidism. This is not a good phenotypic match. ACMG criteria; SYTL5 - VUS- PP3 - Multiple in silicos predict damaging; PAX8 - VUS - PP3 - Multiple in silicos predict damaging.

AH009: Proband 19M. A de novo nonsense variant was identified on trio GS in *SP1* in an individual with syndromic ID, connective tissue disorder. Features included ID, lymphoedema, scoliosis, leg length discrepancy, bicuspid aortic valve with tortuous aortic arch but no true coarctation, poor dental enamel, recurrent otitis media, difficulties with fine motor skills and social difficulties, mild intention tremor. This is a ubiquitously expressed transcription factor which is known to be intolerant to loss of function. No known human phenotype/disease has yet been linked to this gene. This variant has been added to the gene matcher database. ACMG criteria; Pathogenic -PM2 - Absent from population databases, PS2 - de novo (pat conf), PVS1 - Null variant.

AH010: Proband 39F. Compound heterozygous variants (a nonsense variant and a missense variant) were identified on ES re-analysis in *MFSD12* in an individual with ID, epilepsy, spasticity, dysarthric speech and dyspraxia. These have been added to the Gene Matcher database where other patients with variants in the *MFSD12* gene have been reported (an inconsistent phenotypic match). ACMG criteria; c.835C>T - VUS - PM4 - truncating, PM2 - absent in population; c.299G>T - VUS - PM2 - absent in population (1 individual on ExAC), PP3 - Multiple in silicos predict deleterious effect (SIFT, Polyphen2, Grantham 109, CADD 29.2).

AH014: Proband 69F: A missense variant in the *TTN* gene was identified on WES re-analysis and trio GS in an individual with dilated cardiomyopathy (DCM), progressive myopathy and a maternal history of cardiomyopathy. She had 2 sons with non-syndromic ID. Pathogenic missense variants in *TTN* are associated with DCM. This variant is absent from population databases (gnomAD). Reported twice on ClinVar as a VUS. ACMG classification - VUS- PM2.

AH023: Proband 30F. A duplication was identified on singleton GS (after duo ES re-analysis) at 14q13.2 of approximately 200kb in size in an individual with unilateral renal agenesis and uterine / vaginal agenesis and a paternal family history of renal agenesis and anomalies of Mullerian tract structures. This includes a whole gene duplication of the *PRORP* (KIAA0391) and *PPP2R3C* genes. Pathogenic variants in *PRORP* are associated with the autosomal recessive combined oxidative phosphorylation deficiency 54 syndrome (the phenotype is highly variable). Many patients have early-onset sensorineural hearing loss, sometimes in isolation, and sometimes associated with global developmental delay or primary ovarian failure. Other features may include peripheral hypertonia, seizures, muscle weakness, behavioural abnormalities, and leukoencephalopathy on brain imaging. Serum lactate may or may not be elevated. This was considered to be a poor phenotype match and had an inconsistent pattern of inheritance. Pathogenic variants in *PPP2R3C* are associated with autosomal dominant spermatogenic failure and autosomal recessive gonadal dysgenesis, dysmorphic facies, retinal dystrophy and myopathy. Both conditions were considered to be a poor phenotype match, and the latter had an inconsistent pattern of inheritance.

AH024: Proband 30M. A mosaic missense variant in *TSC1* was identified at a low (3.6% MAF) level in brain tissue after high depth TSC1/2 NGS sequencing and singleton ES of DNA extracted from blood and brain tissue in an individual with multiple focal cortical dysplasia and a 3 cm diameter cafe au lait macule in the left groin. The presence of this variant could not be validated by ddPCR. A potential cause of this patient's suspected diagnosis of TSC has therefore been identified. ACMG - VUS- This variant was absent from gnomAD (PM2) and ExAC databases and predicted to be deleterious by SIFT and Polyphen2 (PP3).

AH029: Proband 71M. A missense variant in *PLA2G6* was identified on singleton GS in an individual with early onset Parkinson disease and a family history of her mother, and her sister also being affected by early onset Parkinson disease. *PLA2G6* is associated with several AR phenotypes: Parkinson disease type 14, Neurodegeneration with brain iron accumulation type 2B and infantile neuroaxonal dystrophy 1.

AH032: Proband 36M and his affected twin brother, father and three sons recruited. A novel STR with an ACAGG motif was identified by quad GS of proband and his three affected sons in the *ZNF37A* gene using the Expansion Hunter de novo algorithm. This was in all four sequenced affected individuals with a diagnosis of AD faciomandibular myoclonus with geniospasm. *ZNF37A* is a krab containing zinc finger protein and is thought to function as a transcriptional repressor. It has not previously been associated with any disease phenotype.

AH040: Proband 35F Homozygous missense variants were detected after trio GS in *ANO5* and *ELMO1* and a de novo heterozygous missense variant in *HEPACAM* were identified in an individual with epilepsy since age 2, ID (IQ50) and consanguineous parents. *ANO5* (anoctamin 5) belongs to the anoctamin family of transmembrane proteins and is involved in intracellular calcium activated chloride channel activity. Among its related pathways are ion channel transport and transport of glucose and other sugars, bile salts and organic acids, metal ions and amine compounds. Autosomal dominant mutations in this gene have been associated with gnathodiaphyseal dysplasia (OMIM# 166260). Autosomal recessive mutations in this gene have been associated with Miyoshi Muscular Dystrophy (OMIM# 613319) and limb-girdle muscular dystrophy-12 (LGMDR12) (OMIM# 611307).

The spectrum of autosomal recessive ANO5 muscle disease (anoctaminopathy) is a continuum that ranges from asymptomatic elevations of creatine kinase in blood and exercise-induced myalgia to proximal and/or distal muscle weakness. Intellectual disability and epilepsy are not known to be features of the condition. It seems likely that the main disease mechanism in anoctaminopathies is loss of function. The variant seen in this patient (NM_213599.3(ANO5):c.155A>G (p.Asn52Ser)) has been reported on ClinVar with conflicting interpretations of pathogenicity (ClinVar Variation ID: 197403). The variant allele frequency on gnomAD is 0.002677 and two homozygotes have been reported. This variant has been reported in the following publications: (PMID: 22980763, 23041008, 26810512, 31931849, 32419263, 25891276, 26911675, 25326637, 23606453, 30564623, 30919934, 31517061). *ELMO1* is a cytoplasmic adapter protein that interacts with the DOCK family. to promote activation of the small GTPase RAC. It is not known to be associated with any disease phenotype. The *ELMO1* variant has been added to the Gene Matcher database. *HEPACAM* is a cell adhesion molecule of the immunoglobulin family. It is associated with autosomal recessive megalencephalic leukoencephalopathy with subcortical cysts 2A and autosomal dominant megalencephalic leukoencephalopathy with subcortical cysts 2B +/- intellectual disability. This is not a good phenotype match for our patient. All 3 variants are classified as VUSs according to ACMG criteria.

AH042: Proband 53F. Ataxia with affected maternal cousin. Singleton GS detected an inframe insertion of 13* GCT repeats in an STR downstream of the *ATXN3* gene. Pathogenic CAG repeat expansions in an intragenic STR in *ATXN3* are associated with AD Machado-Joseph disease. There is no known phenotype linked to this GCT STR.

AH043: Proband 56F. A homozygous splice donor site variant was identified on singleton GS in the *RECQL4* gene in an individual with a personal history of three primary malignancies (thyroid, melanoma and ductal carcinoma in situ), xanthoma disseminatum and diabetes mellitus.

Heterozygous / homozygous pathogenic variants in the *RECQL4* gene are associated with several phenotypes: Baller-Gerold syndrome (craniosynostosis and radial aplasia), RAPADILINO syndrome (radial/patellar aplasia/hypoplasia) and Rothmund-Thompson syndrome type 2 (poikiloderma, congenital bone defects, increased risk osteosarcoma in childhood and skin cancer in adulthood). ACMG criteria- VUS- PM2 - Absent in controls. Effect on splicing unknown - RNA studies not undertaken.

AH047: Proband 35F. A 4kb heterozygous intronic deletion (? affecting splicing) of *LARGE1* was identified on singleton GS in an individual with progressive cerebral atrophy, cognitive impairment and epilepsy. Biallelic pathogenic variants in *LARGE1* are associated with the muscular dystrophy-dystroglycanopathy syndrome (Intellectual disability, congenital brain and eye anomalies). This is not a good clinical fit for the patient’s phenotype.

AH048: Proband 25M. A pathogenic frameshift variant (maternal) in *SLC25A15* and a duplication (paternal) of 13q14.11 involving *SLC25A15* were identified on trio GS in an individual with- dysmorphic facies, ID, epilepsy, absent speech, abnormal gait, seminoma, DVTs, polymicrogyria.

Biallelic pathogenic variants in *SLC25A15* are associated with the Hyperornithinemia-Hyperammonemia-Homocitrullinuria (HHH) syndrome. Clinical features may include; chronic neurocognitive deficits, acute encephalopathy secondary to a hyperammonemic crisis and chronic liver dysfunction. The severity of the condition is highly variable. Metabolic studies were normal (did not confirm HHH) and dysmorphic facial features are not in keeping with HHH.

AH049: Proband 35F. A triplication of 2p22.2 containing 22 genes including *EIF2AK2* was identified on singleton GS in an individual with microcephaly, ID, scoliosis and orthopaedic problems (Mowat-Wilson like phenotype). No comparable CNVs are present on the Decipher database. Pathogenic variants in *EIF2AK2* are associated with autosomal dominant and recessive dystonia and the autosomal dominant leukoencephalopathy, developmental delay, and episodic neurologic regression (LEUDEN) syndrome. There was not a good phenotype match but as a triplication a plausible candidate variant.

# **Appendix 3 - Summary of unsolved cases**

AH003: 2 sisters 41,43 recruited with nephrotic syndrome (1 further affected sibling and 1 unaffected sibling). Proband also has mild optic atrophy and idiopathic intracranial hypertension. No personal or family history of hearing impairment or Alport related eye disease: ES re-analysis and QUAD GS (probands and parents) - No candidate variants.

AH011: Proband 60M. Idiopathic end stage renal failure and significant proteinuria necessitating dialysis / transplant in three siblings: Singleton ES re-analysis - no candidate variants found.

AH012: Proband 58F with brittle bone disease (?OI) (minimal trauma fractures of pubic bone, fingers, vertebrae) with tumoral calcinosis (elevated PTH with soft tissue calcification). A brother had bilateral hip replacement aged 30, had spinal fusion aged 26 and multiple clavicular fractures, a sister was diagnosed with osteoporosis at age 40. Singleton ES re-analysis - no candidate variants found.

AH013: Proband 49M. DCM + FH Sudden cardiac death (maternal grandfather age 56) :Singleton ES re-analysis - No candidate variants.

AH017: Proband 45M and brother 46M diagnosed with multiple sclerosis in their 20s.Familial MOG antibody-positive demyelinating disorder. Daughter also affected age 6 with neuromyelitis optica (recruited to RDNow study RCH). Quad GS (proband, brother, parents) - No candidate variants identified.

AH019: Proband 43M: ES re-analysis and singleton GS in individual with epilepsy, ID and Marfanoid systemic features.

AH020: Proband 47M. Febrile seizure. Foci of cortical dysplasia consistent with multiple tubers in left hemisphere, cluster facial angiofibromas, periungual fibroma, hypopigmented lesion : TSC analysis (deep sequencing and ddPCR) - No candidate variants identified.

AH022: Proband 37F with bilateral renal angiomyolipomas, no other features of TSC. No FH of TSC related conditions: TSC analysis (deep sequencing and ddPCR) - One very low level missense mosaic TSC variant identified on deep sequencing - failed to validate on ddPCR and so not reported here.

AH030: Proband 42M bilateral renal AMLs, probable SEGA, seizures, No FH of features of TSC: TSC analysis (deep sequencing and ddPCR) - No candidate variants identified.

AH034: Proband 46M multiple facial angiofibromas, no other features of TSC. No FH of features of TSC: TSC analysis (deep sequencing and ddPCR) - No candidate variants identified.

AH036: 18F proband with severe ID and spastic quadriparesis. No relevant FH and no familial consanguinity. : trio GS - No candidate variants identified.

AH037: 49F proband clinical diagnosis of PTEN/Cowden syndrome (Lhermitte Duclos, macrocephaly, thyroid lesions, uterine fibroma, facial trichilemmomas, oral mucosal papillomatosis, acral keratosis on both hands). No relevant FH. - PTEN deep sequencing, singleton GS: No candidate variants identified.

AH041: 17F proband syndromic epileptic encephalopathy - tonic clonic seizures, absence seizures, myoclonic seizures, exomphalos, severe ID, ASD, sleep disorder, chronic lung disease, right sided kidney disease, pancreatitis, scoliosis, behavioural problems, exompholos: Array, ES reanalysis, trio WGS - No candidate variants.

AH044: 37F proband hereditary geniospasm (deceased father also affected): Singleton GS - no candidate variants found.

AH050: 41M proband ID, autism and epilepsy. EEG - frontocentral sharp slow discharges: singleton GS - No candidate variants identified.

AH051: 30M proband mild- mod ID, epilepsy, choreiform/dystonic movements, absent speech, anxiety, kyphoscoliosis, aggression, obesity, seizures. Brother has ID, autism, epilepsy, behavioural issues (no movement disorder): Singleton GS - No candidate variants identified.

1. Rafehi H, Szmulewicz DJ, Pope K, Wallis M, Christodoulou J, White SM, et al. Rapid Diagnosis of Spinocerebellar Ataxia 36 in a Three-Generation Family Using Short-Read Whole-Genome Sequencing Data. Mov Disord Off J Mov Disord Soc. 2020 Sep;35(9):1675–9.

2. MaxEntScan::scoresplice [Internet]. [cited 2022 Aug 25]. Available from: http://hollywood.mit.edu/burgelab/maxent/Xmaxentscan_scoreseq.html

3. Reese MG, Eeckman FH, Kulp D, Haussler D. Improved splice site detection in Genie. J Comput Biol J Comput Mol Cell Biol. 1997;4(3):311–23.

4. Splice site analysis - SSF [Internet]. [cited 2022 Aug 25]. Available from: http://www.umd.be/searchsplicesite.html

5. Ye Z, Lin S, Zhao X, Bennet MF, Brown NJ, Wallis M, et al. Mosaicism in Tuberous Sclerosis Complex – Lowering the Threshold for Clinical Reporting. Hum Mutat [Internet]. [cited 2022 Sep 1];n/a(n/a). Available from: https://onlinelibrary.wiley.com/doi/abs/10.1002/humu.24454

6. ClinVar [Internet]. [cited 2022 Aug 25]. Available from: https://www.ncbi.nlm.nih.gov/clinvar/

7. LOVD - An Open Source DNA variation database system [Internet]. [cited 2022 Aug 25]. Available from: https://www.lovd.nl/

8. ESP Databases [Internet]. [cited 2022 Aug 25]. Available from: https://www.embedthis.com/esp/doc/users/database.html

9. gnomAD [Internet]. [cited 2022 Aug 25]. Available from: https://gnomad.broadinstitute.org/

10. Bah I, Fahiminiya S, Bégin LR, Hamel N, D’Agostino MD, Tanguay S, et al. Atypical tuberous sclerosis complex presenting as familial renal cell carcinoma with leiomyomatous stroma. J Pathol Clin Res. 2018 Jul;4(3):167–74.

11. Suspitsin EN, Yanus GA, Dorofeeva MY, Ledashcheva TA, Nikitina NV, Buyanova GV, et al. Pattern of TSC1 and TSC2 germline mutations in Russian patients with tuberous sclerosis. J Hum Genet. 2018 May;63(5):597–604.

12. Palencia-Campos A, Aoto PC, Machal EMF, Rivera-Barahona A, Soto-Bielicka P, Bertinetti D, et al. Germline and Mosaic Variants in PRKACA and PRKACB Cause a Multiple Congenital Malformation Syndrome. Am J Hum Genet. 2020 Nov 5;107(5):977–88.

13. Zhang T, Wallis M, Petrovic V, Challis J, Kalitsis P, Hudson DF. Loss of TOP3B leads to increased R-loop formation and genome instability. Open Biol. 2019 Dec;9(12):190222.

14. Kuipers DJS, Mandemakers W, Lu CS, Olgiati S, Breedveld GJ, Fevga C, et al. EIF2AK2 Missense Variants Associated with Early Onset Generalized Dystonia. Ann Neurol. 2021;89(3):485–97.

15. Magrinelli F, Moualek D, Tazir M, Pacha LA, Verghese A, Bhatia KP, et al. Heterozygous EIF2AK2 Variant Causes Adolescence-Onset Generalized Dystonia Partially Responsive to DBS. Mov Disord Clin Pract. 2022;9(2):268–71.

16. Musacchio T, Zech M, Reich MM, Winkelmann J, Volkmann J. A Recurrent EIF2AK2 Missense Variant Causes Autosomal-Dominant Isolated Dystonia. Ann Neurol. 2021;89(6):1257–8.

17. Cagnoli C, Mariotti C, Taroni F, Seri M, Brussino A, Michielotto C, et al. SCA28, a novel form of autosomal dominant cerebellar ataxia on chromosome 18p11.22–q11.2. Brain. 2006 Jan 1;129(1):235–42.

18. Cagnoli C, Stevanin G, Brussino A, Barberis M, Mancini C, Margolis RL, et al. Missense mutations in the AFG3L2 proteolytic domain account for ∼1.5% of European autosomal dominant cerebellar ataxias. Hum Mutat. 2010;31(10):1117–24.

19. Nibbeling EAR, Duarri A, Verschuuren-Bemelmans CC, Fokkens MR, Karjalainen JM, Smeets CJLM, et al. Exome sequencing and network analysis identifies shared mechanisms underlying spinocerebellar ataxia. Brain. 2017 Nov 1;140(11):2860–78.
